# Hepatic granulomas associated with brucellosis: Hepatic granulomas and brucellosis

**Published:** 2011-01-01

**Authors:** Ayse Albayrak, Fatih Albayrak

**Affiliations:** 1 Department of Clinical Bacteriology and Infectious Diseases, Erzurum Region Education and Research Hospital, Erzurum, Turkey; 2 Ataturk University, School of Medicine, Department of Internal Medicine, Division of Gastroenterology, Erzurum, Turkey

**Keywords:** Granulomatous, Hepatitis, Liver, Brucellosis, Biopsy

Brucellosis, a bacterial disease caused by members of the genus Brucella, remains one of the most common zoonotic diseases worldwide [1][2][3]. The disease occurs in both animals and humans, except in those countries where bovine Brucellosis has been eradicated [1]. The bacterial pathogen is classified by the CDC as a category-B pathogen that has potential for development as a bioterrorist agent [1][4]. Brucella spp. is considered to be the most common laboratory-acquired pathogen [1][5]. In humans, Brucellosis behaves as a systemic infection with a very heterogeneous clinical spectrum [2][3][6]. The Brucella organism's predilection for organs rich in reticuloendothelial cells (spleen, liver, bone marrow, lymph nodes) and its intracellular location are responsible for the chronicity of the disease, which can last for months or even years [1][2][7]. Brucella has been reported to compromise the central and peripheral nervous system, as well as the gastrointestinal, hepatobiliary, genitourinary, musculoskeletal, cardiovascular, and integumentary systems [2]. In patients with Brucellosis, the gastrointestinal system is commonly compromised (70%) [5]. Because the liver is the largest organ of the reticuloendothelial system and plays the important role of defense mechanism against Brucella infections, diffuse hepatic involvement is usually recorded during the course of human Brucellosis infection [8]. Brucellosis involves the liver in varying ways, including a slight increase in transaminase levels, mild hepatosplenomegaly, chronic suppurative disease, and, more rarely, acute hepatitis [5][8][9][10][11][12][13]. In patients infected with Brucella melitensis, the involvement of bile canals is observed more often than other Brucellosis factors [14]. Hepatic granulomas are often encountered during liver biopsy and can be caused by a variety of conditions such as a primary hepatic process, fever of unknown origin, or a manifestation of a systemic illness [15][16]. Granulomas are reportedly present in 2 to 10% of all liver-biopsy specimens examined in general practice [17]. Liver Brucelloma, or pseudotumoral necrotizing granuloma, is an uncommon type of hepatic manifestation by Brucella and is observed in only 1.7% of patients affected by Brucellosis [8]. Hepatic Brucelloma is rarely the first to manifest itself clinically, and a focal suppurative lesion may occur if acute Brucellosis is undetected or undertreated in the patient [18]. Granulomas are aggregates of macrophages, often admixed with other inflammatory cells, which usually result from a chronic presence of antigens. Granulomas are a unique inflammatory response that may be idiopathic or may be a response to a bacterial, fungal, viral, or parasitic infection, in the latter cases representing a manifestation of underlying malignancy [15][17].The pathology report on Brucelloma usually shows necrotizing granulomas with a peripheral halo of epithelioid cells. lymphocytes, and plasma cells, as well as and a polimorphonuclear infiltrate in the necrotic area [19][20][21]. Brucellosis involving Brucella abortus is the most common species that can cause hepatic granulomas that are indistinguishable from sarcoidosis [13][16], and Brucella abortus is the most common species that can cause hepatic granulomas. Histopathologically, histiocytic granulomas are present, often with central necrosis, portal and peripheral infiltration, and hyperplasia of the Kupffer cells. [11][22][23]. These granulomas result from the caseation of a granulomatous reaction by persistent Brucellae within macrophages. At present, approximately thirty cases of granulomatous hepatitis (hepatic Brucelloma) have been reported in the literature [19][23][24]. The clinical and biochemical abnormalities return to normal after appropriate treatment [25]. Diagnosis is based on the association of imaging that shows characteristic features (hepatic calcifications) and on positive blood culture and serology [26]. The anti-Brucella Coombs' test titers are especially low. Serological test criteria should be modified for these patients. The detection of calcium densities in the liver is a constant characteristic of hepatic suppurative Brucellosis and reveals the chronic nature of the disease [8]. The histopathological examinations of the US-guided biopsy specimens are not specific, demonstrating only foci of caseous necrosis, or caseous-likesurrounded by an epithelioid granuloma. These findings have also been observed in case studies of patients with tuberculosis, Yersinoios, and Francisella tularensis [18]. The images obtained by US and CT are characteristic. With respect to US, liver Brucelloma has irregular edges and a central or marginal gross calcification. Contrast-enhanced CT shows enhancing trabeculations that separate hypodense solid areas and small liquid collections [19]. In CT scans, calcification with a snowflake appearance may be noted in patients with hepatosplenic abscess. This is a characteristic finding of patients with chronic hepatosplenic supportive Brucellosis and is suggestive of the chronic nature of the disease [8][27]. The presence of a heterogeneous, hypodense lesion is also sometimes observed by an MRI in the T1-weighted sequences and can appear hyperintense in the T2-weighted sequences, at times with an improved demonstration of the saccules containing fluid, which is best seen with MR images [18]. The differential diagnosis of typical Brucelloma must include liver carcinoma, hydatidosis, pyogenic or amebic abscesses, and granulomatous infections, such as tuberculosis and histoplasmosis, which contain similar calcifications [19]. If the lesion is not calcified, the differential diagnosis must include abscesses of different etiologies and primary or metastatic neoplasm [8][19]. The curative therapy in cases of Brucelloma is a combination of medical treatment and percutaneous drainage or surgical resection [3][19][21][24]. The latter is needed because the microorganism can remain within the calcified granulomas, protected from the action of the antimicrobial agent and the host's immune mechanisms [20]. The most appropriate antibiotic combination therapy for patients is not known, but the combination of doxycycline and an aminoglycoside is probably best, and the addition of rifampin may be a reasonable option [8]. The appropriate duration of antibiotic therapy for patients has not been well established, but it seems reasonable to prolong oral therapy for several months [8]. Although several examples of cures after medical treatment have been documented, reactivations are frequent, even months or years later [3][19]. While developing countries are particularly susceptible, the entire world suffers from significant losses related to Brucellosis. Although effective vaccines have been developed for animals, no similarly effective vaccine yet exists to prevent Brucellosis in humans. Therefore, Brucellosis-related public-health issues and economic losses may be minimized by encouraging animal vaccination programs.
